# Monocyte CD36 Expression Predicts Disease Activity in Patients With Crohn's Disease

**DOI:** 10.1155/2024/9202686

**Published:** 2024-11-01

**Authors:** Jiejie Zhu, Nannan Zhu, Jiren Wang, Qiuyuan Liu, Qiao Mei

**Affiliations:** ^1^Department of Gastroenterology, The First Affiliated Hospital of Anhui Medical University, Hefei, China; ^2^Anhui Provincial Key Laboratory of Digestive Disease, The First Affiliated Hospital of Anhui Medical University, Hefei, China

**Keywords:** CD36, Crohn's disease, disease activity, monocyte

## Abstract

**Background:** Crohn's disease (CD) is a chronic intestinal inflammatory disease associated with genetic, environmental, and other unknown factors. Cluster of differentiation 36 (CD36) plays an important role in cancer, inflammation, and metabolic diseases. Although CD36 has recently been implicated in various diseases, its role in CD is still unclear.

**Methods:** Blood samples were collected from patients with CD and healthy volunteers. Peripheral blood mononuclear cells (PBMCs) were isolated by density gradient centrifugation over Ficoll–Paque and labeled with monoclonal antibodies (CD14-APC and CD36-PE). Flow cytometer CytoFlex is used for analysis.

**Results:** Twenty-nine patients with CD in remission, 42 patients with active CD, and 23 healthy volunteers were included in the study. Our results showed that the frequency of the CD14+CD36+ monocyte subset was increased in PBMCs from patients with active CD compared with patients in remission and healthy controls. However, CD36 on monocytes was lower in CD compared with the healthy controls. CD36 expression was decreased in patients with active CD compared with that of patients with CD in remission and healthy control subjects, but no difference was found between patients with CD in remission and healthy controls. Interestingly, we found negative correlations of CD36 with HBI, SES-CD, C-reactive protein, and neutrophil-to-lymphocyte ratio.

**Conclusions:** These data indicate that monocyte CD36 associates with disease activity in CD and might be a potential biomarker for assessing the activity of CD.

## 1. Introduction

Crohn's disease (CD) is a chronic inflammatory bowel disease (IBD) of the gastrointestinal tract. A majority of the patients with CD have been detected and diagnosed during the late teens and early twenties, mostly before age 40 years [1]. Its clinical manifestations are diverse and commonly present with abdominal pain, diarrhea, rectal bleeding, weight loss, and extraintestinal complications, which seriously affect patients' quality of life [2]. Moreover, the incidence of CD is rising, posing important challenges to healthcare systems around the world [3].

CD is an immune-mediated disease characterized by episodes of intestinal and systemic inflammation [4]. Under inflammatory conditions, abundant blood monocytes migrate to the inflamed intestinal mucosa and turn into inflammatory macrophages, which are key players during intestinal inflammation, displaying respiratory burst activity and releasing proinflammatory cytokines, such as interleukin (IL)-1, IL-6, TNF-*α*, and IL-23, in response to inflammatory stimulation [5]. Moreover, intestinal lamina propria monocytes get involved in disrupting the epithelial barrier and inducing apoptosis in epithelial cells, thus driving intestinal inflammation in CD [6].

Cluster of differentiation 36 (CD36), as a transmembrane glycoprotein (GP), is also known as scavenger receptor B2 (SR-B2), fatty acid translocase (FAT), platelet GP IV, and GP88, and it is expressed on the cell surface of multiple cell types, including monocytes, adipocytes, platelets, myocytes, enterocytes, and hepatocytes [7]. Accumulating studies have revealed that CD36 plays vital parts in inflammation and metabolic diseases, including nonalcoholic steatohepatitis [8], atherosclerosis [9], diabetes [10], and cancer [11]. Thus, we hypothesize that monocyte CD36 may have a role in CD.

For now, there are a few studies about the relationship between CD36 and CD. During chronic mucosal inflammation in CD, CD36 was previously reported to be an important receptor in intestinal epithelial cell cycle arrest induced by advanced oxidation protein products [12]. Furthermore, low expression of CD36 was implicated in the damaged mucosa of patients with IBD, regulated by hypoxia-inducible factor-1 [13]. In the peripheral blood of patients with IBD, platelet CD36 expression was elevated compared with healthy subjects, which may contribute to an increased risk of thromboembolism [14]. At present, the value of CD36 expression in CD is controversial and incompletely evaluated. It still needs extensive studies on CD36 in patients with CD. Therefore, this clinical study was designed to explore the expression of monocyte CD36 in patients with CD and its relationship with disease activity.

## 2. Materials and Methods

### 2.1. Patient Enrollment

The study was approved by the Institutional Ethics Committee of Anhui Medical University. Eligible patients and healthy control (HC) subjects signed an informed consent to participate in the study. From January 2023 to May 2023, peripheral blood was obtained from patients with CD who were treated at the Department of Gastroenterology of the First Affiliated Hospital of Anhui Medical University, Hefei, China. All patients were diagnosed with CD based on clinical symptoms, physical examination, endoscopic features, and radiological and histologic evaluation by gastroenterologists. Clinical disease activity was assessed by the Harvey–Bradshaw index (HBI), as follows: remission (≤ 4), mildly active (5–7), moderately active (8–16), and severely active (> 16) [15]. Exclusion criteria were used as follows: (i) pregnant or lactating women; (ii) the patient cases were associated with recent acute gastrointestinal infections or digestive ulcers; (iii) patients with diabetes, hematological diseases, HIV infection, tumors, or other autoimmune diseases; (iv) patients with severe heart, lung, liver, kidney, and other organ dysfunction; (v) the clinical data were incomplete. Clinicopathologic data, including age, sex, level of C-reactive protein (CRP) and erythrocyte sedimentation rate (ESR), fecal calprotectin (FC), hematocrit (Hct), neutrophil-to-lymphocyte ratio (NLR), and platelet count (PLT), were evaluated by reviewing the patients' medical records. The simple endoscopic score for Crohn's disease (SES-CD) is used for endoscopy analyses [16]. No data were imputed for missing values. Meanwhile, blood samples were collected from healthy volunteers as HCs. None of the subjects in the HC group were taking any medication, had infection or inflammatory diseases, or had gastrointestinal diseases. All the research with human subjects was in accordance with the Helsinki Declaration.

### 2.2. Blood Collection and Isolation of PBMC

Peripheral venous blood samples of 3 mL were collected in EDTA tubes from all the enrolled patients with CD and controls. Peripheral blood mononuclear cells (PBMCs) were isolated by density gradient centrifugation over Ficoll–Paque (TBD, Tianjin, China). Immediately after blood collection, blood samples were diluted with the same volume of phosphate-buffered saline (PBS). Subsequently, the diluted cell suspension was carefully layered over Ficoll–Paque without disturbing the interface and then centrifuged at 600 × g for 20 min at room temperature. The PBMCs were collected at the corresponding layers and washed twice with PBS by centrifugation for 10 min at 400 × g.

### 2.3. Flow cytometry

PBMCs were prepared and analyzed by flow cytometry following the manufacturer's instructions. For the cell surface staining, PBMCs were incubated with antibodies (APC anti-human CD14, clone M5E2, Biolegend; PE anti-human CD36, clone 5-271, Biolegend) in the dark for 20 min at room temperature according to the recommendations from the manufacturer. After washing twice, dead cells were then excluded with 4⁣′,6⁣′-diamidino-2-phenylindole (DAPI) (catalog 422801, Biolegend) staining before acquisition on an 8-laser flow cytometer, CytoFlex (Beckman Coulter, San Diego, CA, United States). A minimum of 100,000 events was counted in each sample. Positive and negative gates for each antibody were set with fluorescence-minus-one and blank controls. Specifically, we used one sample and separated it into multiple groups: unstained controls, only staining with DAPI, staining cells with DAPI and CD14, staining with DAPI and CD36, staining with DAPI and CD14, and staining with DAPI, CD14, and CD36. Flow cytometric data were gated and compensated based on the FMO and the empty controls. Double-positive monocyte cells were gated by CD14 and CD36. Gating strategies are shown in Figure S1. Monocyte CD36 expression was reported as mean fluorescence intensity (MFI) (Arbitrary Units). Finally, the analysis of the data was performed using the FlowJo software Version 10.8 (BD Biosciences, United States).

### 2.4. Statistical Analysis

Statistical analysis was performed using SPSS 19.0 edition (IBM Corporation, Armonk, NY, United States) and GraphPad Prism Software 8.0 edition (GraphPad Software, San Diego, CA, United States). Statistical significance was calculated with a Student's *t*-test for comparisons between patients with CD and controls. A Student's *t*-test was also used to evaluate the associations between monocyte CD36 levels and clinicopathologic factors. Correlation between different indexes was analyzed by Spearman's rank correlation test. Data are presented as the mean ± SEM, and the significance level was set at less than 0.05.

## 3. Results

### 3.1. Clinical Features of Patients

A total of 94 subjects were enrolled in this study, which comprised 71 patients with CD and 23 HCs. The characteristics of all the patients are presented in Table 1. The median age of patients with CD was 33 years, and 49 of the patients were male. HCs had a mean age of 30 years, and 10 of them were male. There were statistical differences in gender (*p* = 0.028), but no significant differences in age (*p* = 0.692). Among these patients with CD, the median duration of the disease was 2 years. Participants aged between 17 and 40 years comprised 64.79% of the cohort. CD affected the ileum in 38.03%, the colon in 4.22%, and both the ileum and the colon in 57.75% of cases. As shown in Table 2, there were 42 patients with CD in the active phase, including 30 males and 12 females, with a median age of 35 years; there were 29 patients in the remission phase, including 19 males and 10 females, with a median age of 32 years. No differences were found in age, gender, disease duration, age at first diagnosis, disease location, disease behavior, or previous biological therapy between the two group patients (*p* > 0.05), although significant differences in CRP and FC (*p* < 0.001) were observed between groups. There were also significant differences in HBI score, SES-CD score, or HCT between patients with active CD and patients in the remission phase (*p* < 0.001).

### 3.2. The Frequency of CD14+CD36+ Double-Positive Cells Was Increased in PBMCs From Patients With Active CD

**Figure 1:**
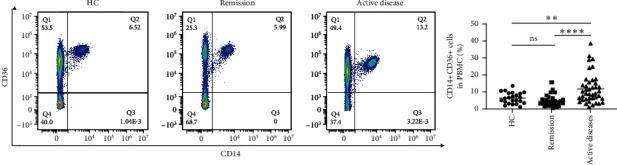
Frequency of CD14+CD36+ double-positive cells in patients with Crohn's disease (CD) and healthy control (HC) subjects. The frequency of CD14+CD36+ double-positive cells in patients with CD in remission (*n* = 29), patients with active CD (*n* = 42), and healthy controls (*n* = 23). The data were presented as the average ± SEM. ⁣^∗∗^*p* < 0.01; ⁣^∗∗∗∗^*p* < 0.0001.

As we all know, monocytes play a vital role in the pathogenesis of CD. Therefore, we first analyzed the percentage of double-positive monocyte cells in peripheral blood from patients with CD and HCs via gating by CD14 and CD36. We found that almost all monocytes expressed CD14, as well as CD36. As shown in Figure 1, the frequency of CD14+CD36+ double-positive cells was increased in PBMCs from patients with active CD than patients in remission (11.74 ± 1.34 vs. 4.81 ± 0.63%, *p* < 0.0001) or healthy controls (11.74 ± 1.34 vs. 6.40 ± 0.68%, *p* < 0.01).

### 3.3. CD36 Expression on Monocytes in Patients With CD and HCs

**Figure 2:**
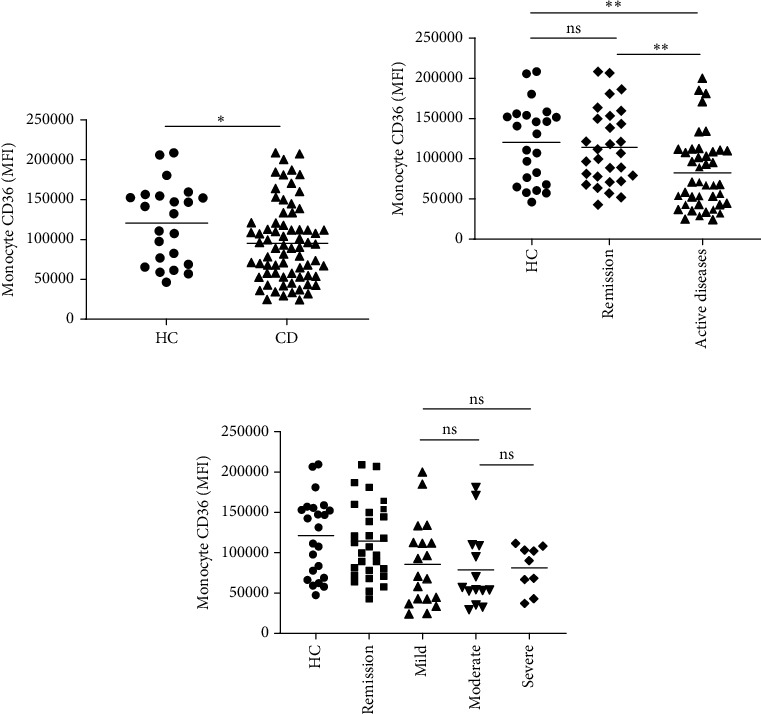
Monocyte CD36 expression (MFI) in patients with Crohn's disease (CD) and healthy control (HC) subjects. (a) The expression of monocyte CD36 was lower in patients with CD compared with HC subjects. (b) CD36 was decreased in patients with active CD compared with patients with CD in remission and healthy controls. (c) No differences in monocyte CD36 were detected between groups with different severities of illness. Patients with CD were classified into the remission (*n* = 29), mild (*n* = 19), moderate (*n* = 14), and severe (*n* = 9) groups according to Harvey–Bradshaw index (HBI). The data were presented as the average ± SEM. ⁣^∗^*p* < 0.05, ⁣^∗∗^*p* < 0.01. MFI, mean fluorescence intensity.

Subsequently, we analyzed the expression of CD36 on peripheral blood monocytes in all patients. The results showed that CD36 on monocytes was lower in CD compared with the control group (95427 ± 5785 vs. 120463 ± 10263, *p* = 0.036) (Figure 2(a)). CD36 expression was decreased in patients with active CD (82356 ± 7092) compared to that of patients with CD in remission (114359 ± 8751, *p* = 0.006) and HC subjects (120463 ± 10263, *p* = 0.003). However, no difference was found between patients with CD in remission and HCs (*p* = 0.651) (Figure 2(b)). No significant difference in CD36 expression was observed between patients with active CD with different severities of illness (*p* < 0.05), as shown in Figure 2(c).

### 3.4. Relationships Between the Expression of Monocyte CD36 and Clinical Indexes

**Figure 3:**
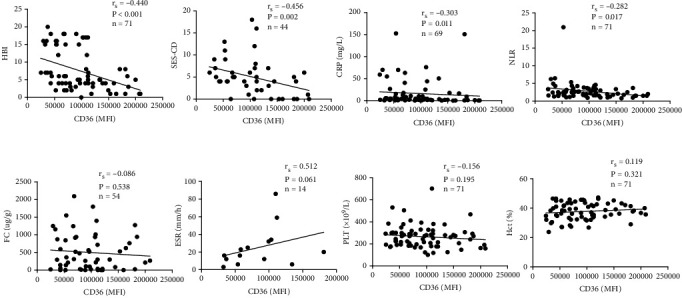
Correlation of monocyte CD36 expression with disease activity and some clinical indexes. (a) There was a negative correlation of the expression of monocyte CD36 with HBI (*n* = 71), SES-CD (*n* = 44), CRP (*n* = 69), and NLR (*n* = 71), respectively. (b) The expression of CD36 on monocytes had no correlation with FC (*n* = 54), ESR (*n* = 14), PLT (*n* = 71), or Hct (*n* = 71). HBI, Harvey–Bradshaw index; SES-CD, the simple endoscopic score for Crohn's disease, CRP, C-reactive protein; NLR, neutrophil-to-lymphocyte ratio; FC, fecal calprotectin; ESR, erythrocyte sedimentation rate; PLT, platelets; Hct, hematocrit.

To explore the relationship between the expression of monocyte CD36 and clinical indexes of patients with CD, we analyzed the correlation of CD36 expression and clinical inflammatory indicators related to CD severity, including HBI, SES-CD, CRP, ESR, FC, NLR, HCT, and PLT. Interestingly, negative correlations of CD36 with HBI (*r* = −0.440, *p* < 0.001), SES-CD (*r* = −0.456, *p* = 0.002), CRP (*r* = −0.303, *p* = 0.011), and NLR (*r* = −0.282, *p* = 0.017) were observed in patients with CD. However, the expression of CD36 had no significant relationship with the levels of FC (*r* = −0.086, *p* = 0.538), ESR (*r* = 0.512, *p* = 0.061), PLT (*r* = −0.156, *p* = 0.195), or Hct (*r* = 0.119, *p* = 0.321), as depicted in Figure 3.

## 4. Discussion

Numerous studies documented that the pathogenesis of CD is complicated, involving several aspects. It is essential to maintain intestinal homeostasis. On the one hand, a lack of innate immunity including deficient phagocyte recruitment may lead to the occurrence of CD. On the other hand, chronic overactivation of the intestinal immune system, including increased cytokine secretion and accumulation of monocytes in the affected areas, constitutes a crucial element for maintaining the disease activity of CD [17, 18]. Smythies et al. [19] observed that intestinal macrophages do not proliferate and also showed that peripheral blood monocytes are the only source of intestinal macrophages. Moreover, several studies have found changes in terms of the composition of monocytes in CD. Thiesen et al. [20] confirmed that the proportion of classical blood monocytes decreased whereas the intermediate monocytes increased. This study showed an increase in the proportion of CD14+CD36+ double-positive cells in PBMC isolated from patients with active CD compared with patients in remission or HCs. However, the roles of the different peripheral blood monocyte subsets in CD are still doubtful and have not been sufficiently evaluated.

Subsequently, we analyzed the expression of CD36 on the surface of monocytes in peripheral blood PBMC. CD36 plays a critical role in the process of inflammation and exerts a proinflammatory effect in many cases, including chronic diseases such as diabetic nephropathy, lupus nephritis, and acute illnesses like acute lung injury [21–23]. Therefore, there has been increasing interest in CD36 as a target to resolve inflammation. Evidence suggests that blockade or deficiency of CD36 can block kidney injury and protect against kidney fibrosis [24]. In hepatic steatosis mice, CD36 deletion reduced liver lipid content and could also contribute to improved insulin sensitivity [25]. However, several studies support the role of CD36 in suppressing inflammation reactions. For example, Ballesteros et al. [26] demonstrated that CD36 expression helped resolution of inflammation through phagocytosis and limited inflammatory tissue injury in stroke. In terms of gastrointestinal disorders, loss of CD36 in endothelial cells impairs stomach function and mucosal renewal after injury, associated with the increasing risk of ulcer, gastritis, and gastrointestinal hemorrhage [27]. Moreover, CD36 deletion results in chronic neutrophil recruitment of the gut, impairs epithelial barrier integrity in the small intestine, and systemically causes subclinical inflammation [28]. These results indicate that CD36 might play an important role in gut inflammatory diseases.

In order to explore this question, we compared the CD36 expression in patients with CD and healthy individuals instead of animals such as mice null for CD36. We observed a significant decrease in monocyte CD36 in peripheral blood PBMC from patients with CD. Previous studies revealed highly upregulated expression of CD36 in circulating monocytes of patients with acute coronary syndromes, infectious diseases like HIV infection and chronic hepatitis B-infected patients with anxiety [29–31]. Nevertheless, our findings are consistent with these studies showing low levels of CD36 in peripheral blood monocytes in patients with rheumatoid arthritis and subclinical atherosclerosis and children with chronic kidney disease [32, 33]. These results demonstrate that inflammation is not always associated with CD36 upregulation and CD36 is able to support both anti and proinflammatory activation. Direct or indirect relationships exist between dysregulation of CD36 and various diseases.

In this study, our results showed no significant difference in CD36 expression between patients with CD in remission and the control group. Compared with HCs and patients in the remission period of CD, the expression of CD36 on peripheral blood monocyte cells of patients with active CD was decreased, but there was no statistically significant correlation with disease severity. SES-CD is a scoring system based on endoscopic variables, including the size of ulcers, surface area covered by ulcers and affected by disease, and presence of stenosis, so as to evaluate the severity of the disease [34]. However, many enrolled patients did not do endoscopy recently, and there is insufficient evidence for endoscopic evaluation. Therefore, the HBI score was adopted in this study to assess the disease activity and severity of all patients with CD. HBI is a simplification of Cronin's disease activity index (CDAI), good and easy to be a widely used measure of clinical status in patients with CD [35]. However, the HBI scoring is subjective to a certain extent, which may be a large part of the reason why the expression of CD36 was not obviously different in patients with different disease severity. Correlation analysis showed that CD36 expression was negatively correlated with HBI and SES-CD score.

This study also investigated the relationship between CD36 expression and systemic inflammatory markers, including CRP, NLR, FC, ESR, Hct, and PLT. These inflammatory markers have been reported to be associated with CD disease activity in a variety of studies [36–38]. In this study, CD36 expression was significantly correlated with CRP and NLR, but not with FC, ESR, PLT, or Hct, which might have been influenced by a not large enough sample size and many other factors involved in inflammation. Furthermore, however, our research only focuses on CD36 expression on CD14, which may have left out other types of monocytes. Consequently, more clinical samples and experiments will be required, and it still needs further research and exploration.

In conclusion, our findings reveal that CD14+CD36+ double-positive cells were increased, whereas CD36 was lowly expressed in monocyte cells from the peripheral blood of patients with active CD. Furthermore, CD36 was negatively correlated with indicators of CD activity, including HBI, SES-CD, and systemic inflammatory markers (CRP and NLR). These results suggest that monocyte CD36 might be a potential biomarker for assessing the activity of CD and a likely target for treatment.

## Figures and Tables

**Table 1:** Baseline characteristics of total patients with CD and healthy controls included in the study.

**Characteristics**	**Patients with CD (** **n** ** = 71)**	**Healthy control subjects (** **n** ** = 23)**	**p**
Age, years (median (P_25_, P_75_))	33 (25–46)	30 (25–52)	0.692
Male/female	49/22	10/13	0.028
Disease duration, years (median (P_25_, P_75_))	2 (1-4)		
Age of diagnosis, *n* (%)			
A1 (≤ 16 years)	4 (5.63)		
A2 (17–40 years)	46 (64.79)		
A3 (> 40 years)	21 (29.58)		
Disease location, *n* (%)			
L1 (ileum)	27 (38.03)		
L2 (colon)	3 (4.22)		
L3 (ileocolon)	41 (57.75)		
L4 (upper gastrointestinal location)	0		
Disease behavior, *n* (%)			
B1 (nonstricturing nonpenetrating)	39 (54.93)		
B2 (stricturing)	22 (30.99)		
B3 (penetrating)	10 (14.08)		
P (perianal disease)	6 (8.45)		
Disease phase, *n* (%)			
Remission	29 (40.85)		
Active disease	42 (59.15)		

**Table 2:** Comparison of clinical characteristics between patients with CD in remission and active period.

**Characteristics**	**Patients in remission (** **n** ** = 29)**	**Patients with active CD (** **n** ** = 42)**	**p**
Age, years (median (P_25_, P_75_))	35 (29.5–42)	32 (23.75–41.25)	1.000
Male/female	19/10	30/12	0.596
Disease duration, years (median (P_25_, P_75_))	2 (2–6)	2 (1–4)	0.198
Age of diagnosis			
A1 (≤ 16 years)	1	3	0.763
A2 (17–40 years)	21	25	
A3 (> 40 years)	7	14	
Disease location			
L1 (terminal ileum)	12	15	0.539
L2 (colon)	2	1	
L3 (ileocolon)	15	26	
L4 (upper gastrointestinal location)	0	0	
Disease behavior			
B1 (nonstricturing nonpenetrating)	18	21	0.156
B2 (stricturing)	5	17	
B3 (penetrating)	6	4	
IJAE_8887524P (perianal disease)	3	3	
Previous biological therapy^a^			
Yes	21	28	0.607
No	8	14	
HBI score (median (P_25_, P_75_))	3 (1.5–4)	8 (6–16)	< 0.001
SES-CD score (median (P_25_, P_75_))^b^	0 (0–2)	6 (4–8.5)	< 0.001
CRP, mg/L (median (P_25_, P_75_))^c^	1.27 (0.50–2.71)	8.49 (4.02–39.02)	< 0.001
FC, *μ*g/g (median (P_25_, P_75_))^d^	36.03 (0-477.00)	594.30 (213.80-1156.00)	< 0.001
NLR (Mean ± SD))	2.06 ± 0.90	3.36 ± 3.09	0.005
PLTs × 10^9^/L (median (P_25_, P_75_))	207.00 (172.00–272.00)	290.50 (226.00–356.00)	< 0.001
Hct, % (mean ± SD)	39.55 ± 4.59	39.57 ± 6.26	0.032

Abbreviations: CD, Crohn's disease; CRP, C-reactive protein; FC, fecal calprotectin; HBI, Harvey–Bradshaw index; Hct, hematocrit; NLR, neutrophil-to-lymphocyte ratio; PLT, platelet; SES-CD, simple endoscopic score for Crohn's disease.

^a^Previous biological therapy includes one or more of the following: infliximab, adalimumab, vedolizumab, and ustekinumab.

^b^There were 14 patients with CD in clinical remission and 13 patients with active CD who did not receive endoscope tests during hospitalization.

^c^There was 1 patient with active CD who did not receive a CRP test during hospitalization.

^d^There were 6 patients with CD in clinical remission and 11 patients with active CD who did not receive FC test during hospitalization.

## Data Availability

The data that support the findings of this study are available on request from the corresponding author upon reasonable request.

## Nomenclature

CD: Crohn's disease
CD36: cluster of differentiation 36
CDAI: Cronin's disease activity index
CRP: C-reactive protein
EDTA: ethylenediaminetetraacetic acid disodium salt
ESR: erythrocyte sedimentation rate
FAT: fatty acid translocase
FC: fecal calprotectin
GP: glycoprotein
HBI: Harvey–Bradshaw index
HC: healthy controls
Hct: hematocrit
HIV: human immunodeficiency virus
IBD: inflammatory bowel disease
IL-1: interleukin-1
IL-23: interleukin-23
IL-6: interleukin-6
PBMC: peripheral blood mononuclear cells
PBS: phosphate-buffered saline
PLT: platelet count
SES-CD: the simple endoscopic score for Crohn's disease
SR-B2: scavenger receptor B2
TNF-*α*: tumor necrosis factor-alpha

## Supporting Information

Supporting Information Additional supporting information can be found online in the Supporting Information section. Figure S1: gating strategies for CD14+CD36+ double-positive cells in PBMCs from patients.
