# Supplementary material for: Monocyte CD36 Expression Predicts Disease Activity in Patients With Crohn's Disease
Source: Gastroenterol Res Pract. 2024 Nov 1;2024:9202686. doi: 10.1155/2024/9202686 (PMC11548947; doi:10.1155/2024/9202686)
Supplement: Supporting Information — Additional supporting information can be found online in the Supporting Information section. Figure S1: gating strategies for CD14+CD36+ double-positive cells in PBMCs from patients. [file 9202686.f1.docx]

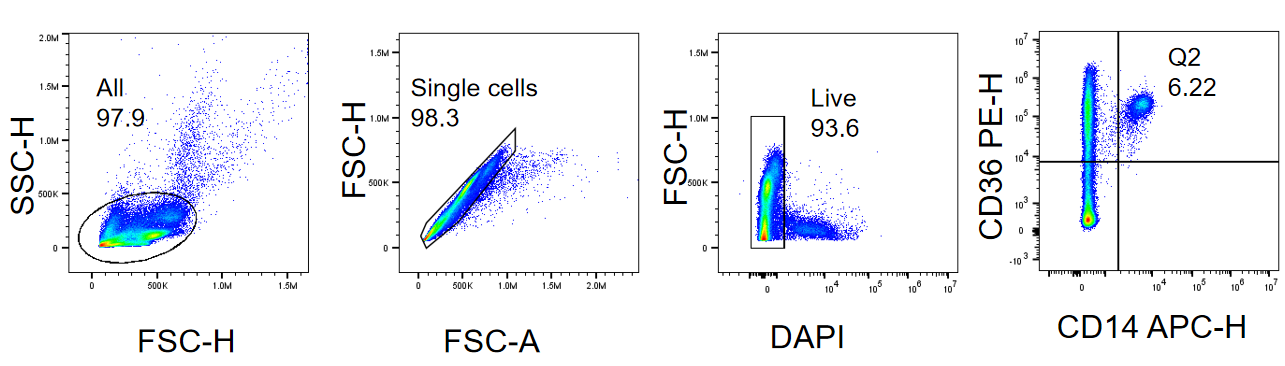


**Supplementary Figure 1: Gating strategies for** **CD14+CD36+ double positive cells in PBMCs from patients.** All cells were identified by their side scatter (SSC-H) and forward scatter (FSC-H) properties. Doublet exclusion was performed by examining the FSC-H vs. FSC-A profile. Live cells were gated on based on their negative expression of DAPI. Finally, the CD14+CD36+ double positive cells were screened by positive CD36 and CD14 expression.
